# Deuterohemin-Ala-His-Thr-Val-Glu-Lys (DhHP-6) Mimicking Enzyme as Synergistic Antioxidant and Anti-Inflammatory Material for Periodontitis Therapy

**DOI:** 10.3390/biomimetics7040240

**Published:** 2022-12-14

**Authors:** Jiaqing Yan, Min Liu, Yan Zhang, Ying Zhu, Qiuyan Chen, Yimeng Yang, Min Hu, Huimei Yu

**Affiliations:** 1 Hospital of Stomatology, Jilin University, Changchun 130021, China; 2 Key Laboratory of Pathobiology, Ministry of Education, Department of Pathophysiology, College of Basic Medical Sciences, Jilin University, Changchun 130021, China

**Keywords:** peroxidase-mimicking enzyme, heme hexapeptide, oxidative stress, periodontitis, reactive oxygen species

## Abstract

Periodontitis is an inflammatory disease induced by plaque microorganisms. In the clinic, antibiotic assistant periodontal mechanical therapy is the most effective therapy for the treatment of periodontitis. However, the drug resistance of the antibiotics and the repeated coming and diminishing of the disorder of oxidation–reduction balance in the inflammatory tissue could not meet the high requirements for periodontic health control in long periods. Deuterohemin-ala-his-thr-val-glu-lys (DhHP-6) is a biomimetic oxidase-mimicking enzyme that simulates the reactive oxygen radical scavenger function of heme by synthesizing the new molecular material following the key structure and amino acid sequence of heme. In this article, we report the antioxidant and anti-inflammatory properties of DhHP-6 by building a inflammatory model for human gingival fibroblasts (HGFs) stimulated by lipolysaccharide (LPS) and its effects on periodontitis in Wistar rats. DhHP-6 reduced the oxidative stress of HGFs by increasing the amount of the reductase species of glutathione (GSH) and catalase (CAT) while decreasing the amount of oxidase species of malonaldehyde (MDA) and reactive oxygen species (ROS). DhHP-6 had a dose-dependent protective effect on alveolar bone absorption in rats with periodontitis, enhanced antioxidant capacity, and reduced inflammation. As determined by Micro-CT scanning, DhHP-6 reduced alveolar bone loss and improved the bone structure of the left maxillary first molar of rats. There were no obvious morphological and histological differences in the rat organs with or without DhHP-6 treatment. These results suggest that DhHP-6 can be used to treat periodontitis by increasing the expression levels of antioxidant enzymes and antioxidants in systemic and local tissues, thereby reducing levels of oxidation products and cyto-inflammatory factors. The synergistic antioxidant and anti-inflammatory effects of DhHP-6 suggest that there are promising applications of this biomimetic enzyme molecular material for the next generation of agents for periodontitis therapy.

## 1. Introduction

Periodontitis is a chronic inflammatory condition associated with a dysbiotic microbial biofilm [1,2]. It can lead to inflammation of the gingival tissue, resulting in the dissolution and destruction of the collagen fibers in the gingival and periodontal membranes, as well as the absorption of the alveolar bone, leading to the loosening and falling out of teeth [3]. Periodontitis is the primary cause of tooth loss in adults. It can destroy the supporting tissues of the teeth and influence systemic health [4].

Increasing research has focused on the effect of oxidative stress, caused by excessive ROS, on the occurrence and development of periodontitis [5]. Vitamin C, an antioxidant, can increase the number of collagen bundles in periodontal tissue, which is beneficial for the treatment of periodontitis [5,6] as antioxidants, resveratrol, and curcumin can improve the antioxidant defense ability of periodontal ligament cells and reduce the inflammatory response in periodontitis [5,7]. However, the maintenance of the oxidative balance requires the continuous input of antioxidants. This strategy resolves oxidative stress but does not address the source of excessive ROS for the curative treatment of periodontitis [8,9].

The most effective way to cure periodontitis is to introduce a new peroxidase into the gingiva and establish a “new” oral redox balance, thereby improving the antioxidant capacity of the whole body, including the local periodontal tissue, to eliminate excess ROS and improve chronic oral infection [10,11,12,13]. Many studies have shown that various antioxidants, such as natural enzymes, mimic enzymes, and natural antioxidants, can effectively maintain the intracellular redox balance and protect cells from oxidative damage [14,15].

Enzymes are biocatalysts (i.e., they accelerate biochemical reactions) and are critical for modulating many essential biological systems. Enzymes contribute to cellular metabolism; their functions are tightly regulated, rapid, and often specific. The human enzymatic system is sensitive to health effects [16,17,18]. An issue with even a single enzyme in an enzymatic system can result in inhibition or inactivation. Depending on the degree and reversibility of the issue, chronic or acute pathological changes or even death can occur. Therefore, to restore biological processes, normal and healthy enzymatic biochemical reactions are required [19,20]. Ensuring the specificity and activity of the relevant enzymatic system is, therefore, a primary therapeutic strategy [21]. DhHP-6, an oxidase-mimicking enzyme developed from microperoxidase-11 [22], has a strong peroxidase activity. It is a novel peroxide-mimicking heme hexapeptide. Its peptide sequence is Ala-His-Thr-Val-Glu-Lys. The proximal histidine can be coordinated with the active central iron to improve its enzymatic activity. It has anti-inflammatory, anti-oxidant, anti-aging, and oxygen radical scavenging functions based on cellular and animal model experiments and, therefore, is an ideal antioxidant enzyme in which DhHP-6 in HepG2 cells can up-regulate the expression of HO-1 protein and the expression of antioxidant enzymes by activating the Nrf2 signaling pathway, showing good antioxidant capacity [23]. Gong et al. found that DhHP-6 showed good antioxidant capacity by improving the activity of peroxidase and directly removing ROS [24]. Therefore, DhHP-6 can not only directly clear intracellular ROS but also activate intracellular antioxidation-related pathways, up-regulate the expression of antioxidant enzymes, and improve the antioxidant capacity of cells. However, the therapeutic effect of DhHP-6 in periodontitis has not been reported.

In this study, a model of oxidative stress in HGFs and a rat model of the periodontitis model were established to determine the antioxidant effects of DhHP-6 and its mechanism of action. The levels of serum oxidant stress markers and inflammatory response markers, as well as the protective effect of DhHP-6 on alveolar bone resorption induced by periodontitis were evaluated. DhHP-6 increases the expression levels of antioxidant enzymes and antioxidants in systemic and local tissues [25] and reduces the levels of oxidation products and cellular inflammatory factors [26]. The results of this study provide valuable evidence supporting the use of DhHP-6 for the treatment of periodontitis.

## 2. Materials and Methods

### 2.1. Cells and Animals

After obtaining informed consent from the patient in the surgery clinic of the Oral Hospital of Jilin University, healthy gingival tissues removed from a teenager during the orthodontic extraction of third molar (No. 38 site according to the Federation Dentaire International System) were collected. The patient had no systemic disease and did not take antibiotics for 2 weeks before surgery. After the gum samples were removed, they were washed immediately with precooled saline and stored on ice. In a biosafety cabinet, the gum samples were cut into small pieces of approximately 0.2 mm × 0.2 mm × 0.2 mm and added to a 25 cm^2^ culture bottle (Corning, New York, NY, USA). Gingival tissues were firstly seeded cultured with Dulbecco’s modified Eagle’s medium (DMEM) containing 10% fetal bovine serum (FBS) and 1% penicillin/streptomycin. After HGFs swim out of the gum tissue, the third generation of HGFs with good growth condition were obtained, digested by 0.25% trypsin and counted. The cells were re-suspended in complete medium, and the cells suspensions were added to 24-well plates for culture, with each group repeating three wells. When the cells grew to 80% ~ 90% abundance, the culture medium was sucked off, the cell slides were taken out, washed with PBS three times for 30 s each time, fixed with 4% paraformaldehyde for 30 min, washed with PBS three times for 30 s each time, and the surface liquid was dried and stored at −20 °C for reserve. Immunohistochemical staining was used for keratin and undulatory filament detection. Male SPF-grade Wistar rats, weighing 200 ± 20 g, were purchased from Changchun Yisi Experimental Animal Co., Ltd. (Jilin, China) and raised at room temperature with freely available food and drinking water.

### 2.2. MTT Assay

An MTT assay was used to detect the effects of different concentrations of DhHP-6 (0, 10, 20, 40, 80, 160, and 320 μM) and H_2_O_2_ (0, 50, 100, 200, 400, 800, and 1600 μM) on the survival rate of HGFs. They were plated on a 96-well plate at 1 × 10^4^ per well and incubated at 37 °C for 24 h. Various concentrations of DhHP-6 and H_2_O_2_ were added for 24 h and each treatment was repeated in three wells. Each well was supplemented with 20 μL of MTT (5 mg/mL) (3-(4,5-dimethylthiazol-2-yl)-2,5-diphenyltetrazolium bromide (Sigma–Aldrich, St. Louis, MO, USA) and incubated for 4 h. Then, 150 μL of dimethyl sulfoxide was added to dissolve the formazan crystals. Absorbance was measured using a Vmax Microplate Reader (Molecular Devices, Sunnyvale, CA, USA) at a wavelength of 570 nm.

### 2.3. ROS, MDA, GSH, and CAT Assays

ROS was measured with 2′,7′-dichloroflurescein-diacetate (DCFH-DA, BestBio, Nanjing, China). Briefly, HGFs were seeded in 24-well plates at a density of 1 × 10^5^ cells/well, with three replicate wells per group, and cultured overnight. Serum-free medium, 20 μM DhHP-6, and 200 μM H_2_O_2_ were added according to the experimental treatment. HGFs pre-treated with 20 μM DhHP-6 for 4 h and then subjected to 200 μM H_2_O_2_ for 20 h were the DhHP-6+H_2_O_2_ group. At the end of the culture period, samples were washed with phosphate-buffered saline (PBS), 1 mL of 10 μmol/L DCFH-DA was added to each well, and incubation was continued for 20 min at 37 °C in dark. PBS was used to wash the cells three times to remove DCFH-DA that did not enter the cells. After 20–30 min, the cells were observed under an IX71 microscope (Olympus, Tokyo, Japan) and images were obtained. MDA content (Wanleibio), GSH concentration, and CAT activity (Solarbio Science & Technology, Beijing, China) were measured following the manufacturer’s instructions.

### 2.4. Analysis of Cell Apoptosis

After exposure to different experimental conditions, HGFs were trypsinized and incubated with propidium iodide (PI) and Annexin V-FITC (Invitrogen, Carlsbad, CA, USA) for 15 min at 37 °C. Apoptosis was analyzed using a CytoFLEX flow cytometer (Beckman Coulter Life Sciences, Indianapolis, IN, USA). Caspase-3, -8, and -9 activity levels were detected by caspase-3, -8, -9 activity assay kits (BestBio, Nanjing, China), according to the manufacturer’s instructions. Briefly, after being lysed on ice for 30 min, different groups of cellular proteins were incubated in reaction buffer with Ac-DEVD-pNA at 37 °C for 4 h. The 405 nm absorbance was measured via NanoDrop 2000 apparatus (Thermo Fisher Scientific, Waltham, MA, USA).

### 2.5. ELISA Assays

When the HGFs reached 80% to 90% abundance at the bottom of the culture bottle, trypsin digestion was centrifuged and cells count was performed. A total of 1 × 10^6^ cells/well were inoculated into a six-well plate, and each group had 3 complex Wells. The supernatant was removed overnight, and LPS 100 μL with a final concentration of 10 μg/mL was added to each well overnight. DhHP-6 with a final concentration of 20 μM was added as required to continue the culture for 24 h. The supernatant was collected, labeled, and stored at −80 °C. IL-1β and TNF-α levels in the supernatant were measured according to the manufacturer’s instructions of Solarbio Science & Technology (Beijing, China).

### 2.6. Rat Periodontitis Model

In total, 50 SPF-grade male Wistar rats, were selected to establish the animal model of acute periodontitis induced by ligation. The rats were kept in a temperature-controlled room (23–25 °C) with alternating light and dark cycles, and fresh drinking water was supplied every day. After 1 week of adaptive feeding, 8 rats were randomly selected as the control group. The rats were anesthetized with 14% isoflurane inhalation anesthesia and no operation was performed to exclude the effect of anesthesia. The other 42 rats underwent surgery according to the following steps. (1) Rats were anesthetized according to the anesthesia method used for the control. After successful anesthesia, the rats were fixed in the supine position on the operating table. (2) The oral cavity was disinfected with 75% ethanol. In the space between the first and the second molar, an orthodontic ligature wire with a diameter of 0.25 mm was passed through the buccal side to the palatal side, around the second molar, and tied. The ligature wire was placed under the gingival sulcus as far as possible to prevent it from falling off and affecting the feeding. (3) The stability of the ligature wire was checked every day during the experiment, and the induction time was one week. At one week after modeling, the rats were examined, according to the clinical diagnostic criteria for periodontitis: (1) the color, shape, and texture of the gums; (2) periodontal bleeding on probing or not; (3) deep periodontal pocket formation by periodontal probing; (4) attachment loss, distinguishing between periodontitis and gingivitis. In accordance with the above four standards, the model was successfully established.

Detection of indexes in rats with periodontitis. Thirty-two successfully modeled rats were selected for the experimental treatment and were randomly divided into four groups (8 rats per group), and there were five total groups, including the control. First, the rats were anesthetized with 14% isoflurane inhalation anesthesia, and the same volume of drugs was injected into each group as follows. In the control group, 20 μL of PBS was injected into the gingiva of the teeth. In the periodontitis group, 20 μL of PBS was injected into the gingiva of model teeth. In the low-dose DhHP-6 group, 20 μL of DhHP-6 in PBS at 0.3 mg/kg was injected into the gingiva of model teeth. In the medium-dose DhHP-6 group, 20 μL of DhHP-6 in PBS at 1.0 mg/kg was injected into the gingiva of model teeth. In the high-dose DhHP-6 group, 20 μL of DhHP-6 in PBS at 3.0 mg/kg was injected into the gingiva of the model teeth. The drug was administered regularly every day for 2 weeks. The rats were anesthetized by isoflurane inhalation, fixed in the supine position, and the oral cavity was photographed. The whole blood was obtained from the heart and centrifuged at 4 °C and 3000 r/min for 10 min. The upper serum layer was collected, labeled, and preserved until the detection of antioxidant and inflammatory factors.

The rats were euthanized by inhaling carbon dioxide, and the heart, liver, spleen, lungs, and kidneys were washed repeatedly with normal saline and fixed in a 10% neutral formalin buffer solution. In all experimental rats, the maxillary molar area on the model side was removed. In four randomly selected rats in each group, the gums around the alveolar bone were peeled off to detect antioxidant and inflammatory factors in the gingival tissue. In the other four rats, samples were taken from the complete maxillary bone (with gums), rinsed with normal saline and fixed in a 10% neutral formalin buffer solution for Micro-CT. Finally, the rat carcasses were collected and handled appropriately. The following parameters were evaluated: (1) gingival color, shape, and texture; (2) body weight; (3) alveolar bone resorption; (4) expression levels of the antioxidant factors GSH, CAT, and SOD in gingival tissues; (5) expression levels of the antioxidant factors GSH, CAT, and SOD in rat serum; (6) expression levels of the serum inflammatory mediators TNF-α and IL-1β; (7) liver function; (8) alveolar bone features determined by micro-computed tomography (Micro-computed tomography, Micro-CT).

### 2.7. Micro-CT Assays

After the rats in each experimental group were killed, the left maxillary specimens were obtained and scanned by Micro-CT to evaluate the extent of alveolar bone loss and the degree of bone destruction. The alveolar bones of rats in each group were taken and scanned by Micro-CT. To ensure the consistency of measurement standards, the visual angles of all images were adjusted so that all tips were on the same plane and the occlusal plane could not be seen from the buccal and palatal sides. Three-dimensional images were reconstructed to quantify the degree of bone destruction. Alveolar bone loss (ABL) was measured along the root axis of the left maxillary first molar. The vertical distance from the cementum enamel junction (CEJ) to the alveolar bone crest (ABC) was measured as an indicator of the degree of alveolar bone loss. The lengths of six anatomic sites were measured, including three sites on the palatal side (proximal, middle, and distal) and three sites on the buccal side (proximal, middle, and distal).

In each sample, the alveolar bone around the maxillary first molar was selected as the region of interest (ROI) to study. The anterior boundary of the ROI was the mesial root part of the left maxillary first molar, the posterior boundary was the mesial root part of the left maxillary second molar, and the upper boundary was the bottom line of the root bifurcation between the first molar and the second molar. The lower boundary was the mesiodistal apical line of the first molar. The following bone microstructural parameters were analyzed in the ROI: bone volume (BV), total volume (TV), bone volume fraction (BV/TV), trabecular number (Tb.N), trabecular thickness (T.Th), and trabecular spacing (Tb.Sp).

### 2.8. H&E Staining Assays

The heart, lung, liver, spleen, and kidney of rats were fixed for 24 h and washed with deionized water. The tissue was routinely treated, dried, and embedded in paraffin; sectioned with a thickness of 4 μm; stained with hematoxylin and eosin (H&E); and examined under a light microscope. The maxillary bone was decalcified in 10% ethylenediamine tetraethyl acid (EDTA) solution for 2 months, dehydrated with ethanol, embedded in paraffin, and sliced (section thickness was 3.0 μm). The attachment loss and alveolar bone resorption were observed under microscope by conventional HE staining and neutral resin seal.

### 2.9. Statistical Analysis

Experiments were performed in triplicate. Results are presented as means ± SD. Groups were compared using two-tailed Student’s *t*-tests implemented in SPSS13.0. Values of *p* < 0.05 were considered statistically significant.

## 3. Results

### 3.1. Effects of DhHP-6 on HGFs Treated with H_2_O_2_

**Figure 1:**
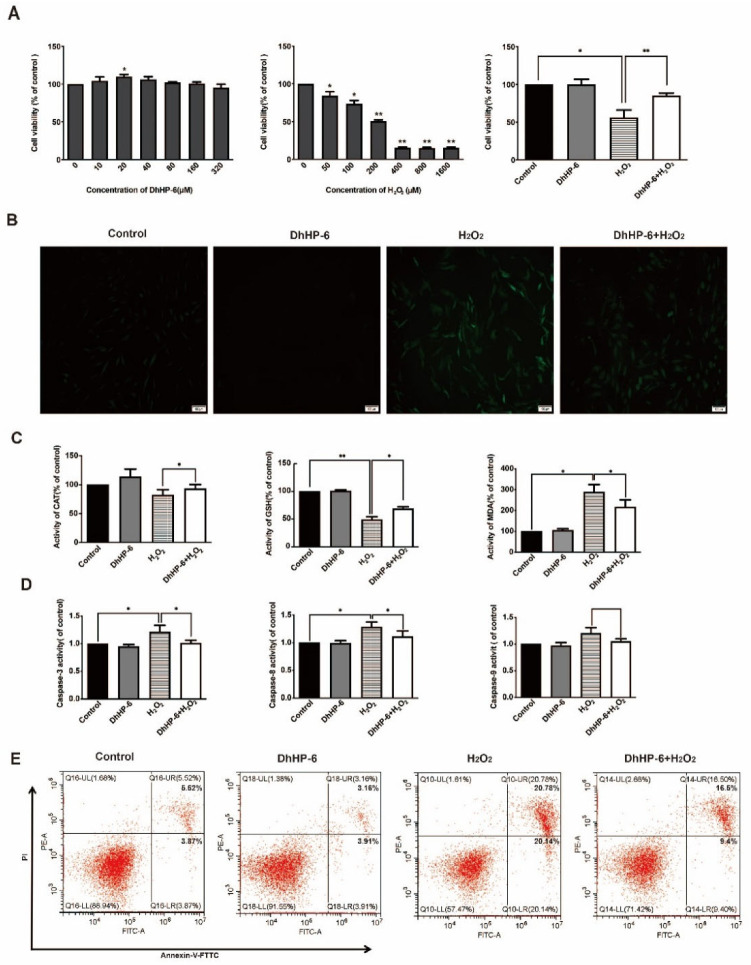
Effects of DhHP-6 on HGFs treated with H_2_O_2_. (**A**) An MTT assay was used to evaluate the proliferation of HGFs treated with DhHP-6 and H_2_O_2_ separately and in combination. (**B**) DCFH-DA staining was used to observe ROS levels in HGFs under different treatment conditions. (**C**) ELISA was used to detect the expression levels of CAT, GSH, and MDA in HGFs treated with DhHP-6 and H_2_O_2_. (**D**) The activity of caspase family members in HGFs treated with DhHP-6 and H_2_O_2_. (**E**) Apoptosis was detected by FACS in HGFs treated with DhHP-6 and H_2_O_2_. (* *p* < 0.05, ** *p* < 0.01).

DhHP-6 reduced oxidative stress damage in human gingival fibroblasts. According to our previous study, the enzyme mimic DhHP-6 has a significant antioxidant activity [27]. To analyze the effect of DhHP-6 on HGFs induced by H_2_O_2_, cell viability was measured by an MTT assay. As shown in Figure 1A, DhHP-6 not only showed no cytotoxicity to HGFs but also promoted the proliferation of HGFs at 20 μM. H_2_O_2_ inhibited cell growth in a dose-dependent manner. The cells survival rate of 200 μM H_2_O_2_ group was significantly decreased and showed obvious cytotoxicity to HGFs cells. The cell survival rate was only 50.80 ± 2.3%, which was significantly lower than that of blank control group. Treatment with 20 μM DhHP-6 pretreatment group could significantly increase the survival rate of HGFs cells damaged by H_2_O_2_, and the difference was significant, compared with H_2_O_2_ group. The results showed that DhHP-6 had a protective effect on H_2_O_2_-induced HGFs cells.

DCFH staining was used to detect cellular ROS levels [28]. Almost no green fluorescence was detected in the cells of blank control group and DhHP-6 group. H_2_O_2_ group produced a large number of ROS in cells, and the green fluorescence intensity was enhanced. A small number of ROS were produced in DhHP-6 + H_2_O_2_ group, and weak green fluorescence was observed, and the fluorescence intensity was significantly lower than that in the H_2_O_2_ group (Figure 1B). The results indicate that DhHP-6 can remove excess intracellular ROS and reduce intracellular ROS accumulation, thus reducing the damage and destruction of cells caused by ROS. The levels of CAT and GSH in HGFs treated with DhHP-6 + H_2_O_2_ were significantly higher than those in HGFs treated with H_2_O_2_ alone. However, the MDA content was significantly reduced, suggesting that DhHP-6 can eliminate excessive intracellular ROS and reduce intracellular ROS accumulation, thereby reducing cell damage. (Figure 1C).

The activation of caspase family proteins is an important sign of apoptosis. Therefore, an activity assay was used to detect the effect of DhHP-6 on HGFs [29]. As shown in Figure 1D, the activity levels of caspase-3, caspase-8, and caspase-9 in cells treated with DhHP-6 + H_2_O_2_ were significantly lower than those in cells treated with H_2_O_2_ alone. As determined by flow cytometry, the rate of apoptosis was significantly lower in DhHP-6 + H_2_O_2_-treated cells than in H_2_O_2_-treated cells (Figure 1E). It indicated that H_2_O_2_ activated caspase-8 and 9 in HGFs, started the external and internal apoptotic pathways of HGFs, and finally activated caspase-3 to make apoptosis enter the irreversible stage. DhHP-6 can effectively reduce the activity of caspase-8 and caspase-9 in HGFs, thereby reducing the activity of caspase-3 and the apoptosis rate of HGFs.

### 3.2. Effects of DhHP-6 on the Expression of HGFs Inflammatory Cytokines Induced by LPS

**Figure 2:**
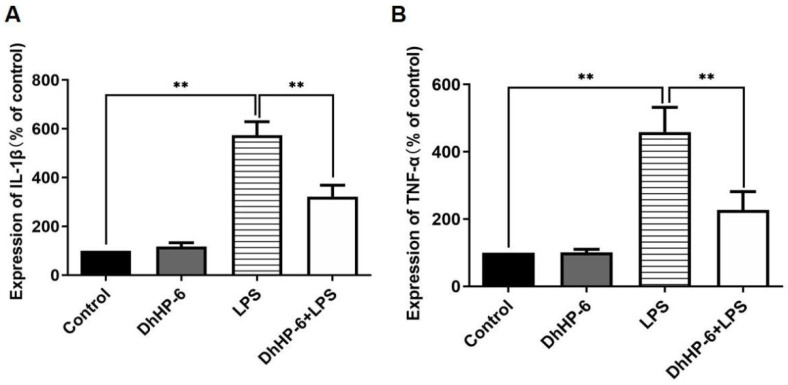
Expression levels of TNF-α and IL-1β in the supernatant of HGFs induced by LPS. (**A**) IL-1β levels in the cell supernatant; (**B**) TNF-α levels in the cell supernatant ** *p* < 0.01, compared with the group treated with H_2_O_2_ and control.

An enzyme mimic reduced the expression of inflammatory factors in human gingival fibroblasts. The pro-inflammatory cytokines TNF-α, IL-1β, and IL-6 play key roles in the occurrence and development of periodontitis [30]. As shown in Figure 2A, the content of IL-1β in the LPS group was significantly higher than that in the blank control group. The content of IL-1β in the DhHP-6 + LPS group was significantly lower than that in the LPS group. As shown in Figure 2B, the content of TNF-α in the LPS group was significantly higher than that in the blank control group. The expression of TNF-α in the DhHP-6 + LPS group was significantly lower than that in the LPS group. These results showed that DhHP-6 reduced the content of inflammatory factors TNF-α and IL-1β in LPS-sensitized HGFs and alleviated the inflammatory response of cells.

### 3.3. The In Vivo Effect of DhHP-6 on Periodontal Histomorphology and Histology

**Figure 3:**
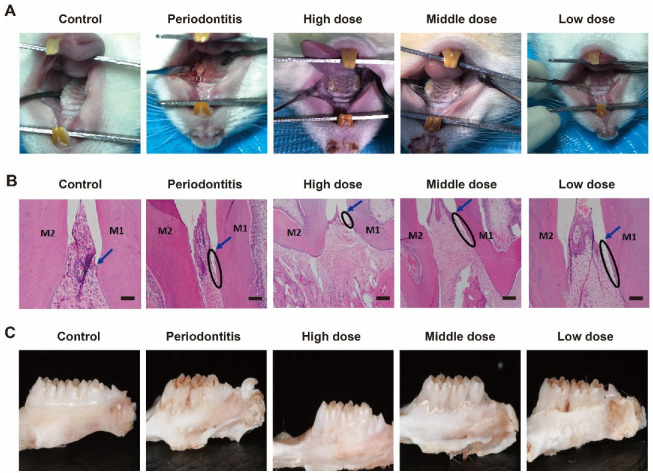
H&E staining and oral conditions of the periodontium 2 weeks after treatment. (**A**) Oral conditions of rats in different groups; (**B**) H&E staining. M1, the first molar; M2, the second molar; Arrow, cementum enamel; ellipse, attachment loss; bar = 100 μm. (**C**) Morphological changes in the alveolar bone of rats in different groups.

Mimic enzymes alleviated periodontitis in rats. DhHP-6 was injected into the gingiva of the model teeth to analyze its therapeutic effects. The rat model teeth formed periodontitis; the gingiva and tooth surface did not adhere to each other, as observed by the naked eye; deeper periodontal pockets appeared; there was a large accumulation of food debris in the periodontal pockets; the gingiva was red and swollen; and it was easy to bleed when probed, which proved that the model was successfully established. With an increase in the DhHP-6 dose, the degree of gingival inflammation was gradually reduced and was positively correlated with the dose. The results demonstrated that DhHP-6 can improve the gingival inflammatory response caused by periodontitis and has a certain therapeutic effect on periodontitis, as shown in Figure 3A. As determined by H&E-stained images of the periodontium 2 weeks after treatment, in the control group, the gingival papilla filled the interdental space, the gingival tissue structure was normal, the bonded epithelium was attached at the cemento-enamel junction, and the periodontal fibers were normally aligned. In the periodontitis and low-dose groups, the structure of the gingival papillae was destroyed, the position of the bound epithelium was significantly displaced towards the roots, deep periodontal pockets formed, and the periodontal membrane fibers were disorganized or broken. Compared to corresponding features in the periodontitis group, the gingival papilla structures were less damaged and shallower pockets were formed in the high- and medium-dose groups, as shown in Figure 3B.

The change in alveolar bone height is an important clinical manifestation of periodontitis [31]. After collecting the rat alveolar bone and peeling away the surrounding gingival tissue, thereby completely exposing the alveolar bone, changes in the height of the alveolar bone were visually observed. Following DhHP-6 treatment with different doses, the alveolar bone was still absorbed. However, compared with the height in rats with untreated periodontitis, the alveolar bone height was significantly reduced and the periodontitis symptoms improved in rats treated with DhHP-6, as shown in Figure 3C. These findings show that DhHP-6 has a dose-dependent protective effect on the absorption of the alveolar bone in rats with periodontitis.

### 3.4. The In Vivo Effect of DhHP-6 on the Expression of GSH, SOD, CAT and Inflammatory Factors in Rat

**Figure 4:**
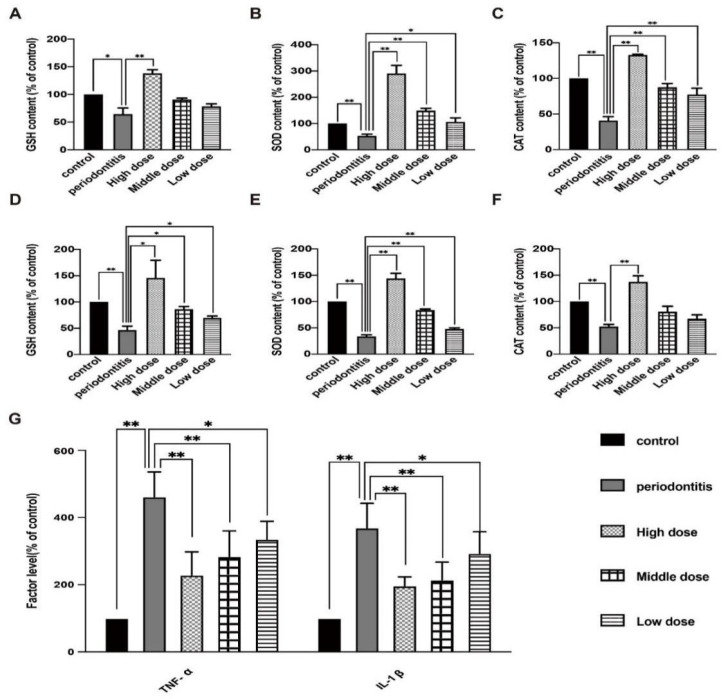
Effect of DhHP-6 on the antioxidant level and inflammation levels in rats. (**A**–**C**) Expression levels of SOD, GSH, and CAT in rat serum. (**D**–**F**) Expression levels of GSH, SOD, and CAT in rat gingival tissues. (**G**) Levels of the inflammatory factors TNF-α and IL-1β in rat serum. * *p* < 0.05, ** *p* < 0.01.

DhHP-6 enhanced the antioxidant capacity and reduced inflammation in rats. To observe the effect of DhHP-6 on antioxidant factors, the expression levels of GSH, SOD, and CAT in the serum and gingival tissues of rats with periodontitis were measured. As shown in Figure 4A–C, the serum levels of GSH, SOD, and CAT in rats with periodontitis were lower than those in untreated rats. With an increase in the DhHP-6 concentration, the GSH, SOD, and CAT levels increased gradually. GSH, SOD, and CAT levels in the high-dose DhHP-6 group were significantly higher than those in the periodontitis group. These results showed that DhHP-6, as a peroxidase-mimic, increased the expression levels of antioxidant factors in local gingival tissue and serum in a dose-dependent manner (Figure 4D–F). Therefore, DhHP-6 can increase the expression of antioxidant factors and enhance the antioxidant capacity at sites of inflammation, maintain tissue redox homeostasis, and reduce oxidative damage in inflammatory tissues under oxidative stress.

Our next goal was to observe the effect of DhHP-6 on periodontitis-related inflammation. ELISA was used to detect the effect of DhHP-6 on the expression of TNF-α and IL-1β in the serum of rats in each group [32]. The results are summarized in Figure 4G. The serum levels of TNF-α and IL-1β in the periodontitis group were higher than those in the blank control group. As the dose of DhHP-6 increased, the levels of TNF-α and IL-1β were significantly lower than those in the periodontitis group. These results showed that DhHP-6 reduced the levels of TNF-α and IL-1β in the serum of rats with periodontitis, improved the inflammatory response, and slowed alveolar bone resorption.

### 3.5. The In Vivo Effect of DhHP-6 on Alveolar Bone in Rats

**Figure 5:**
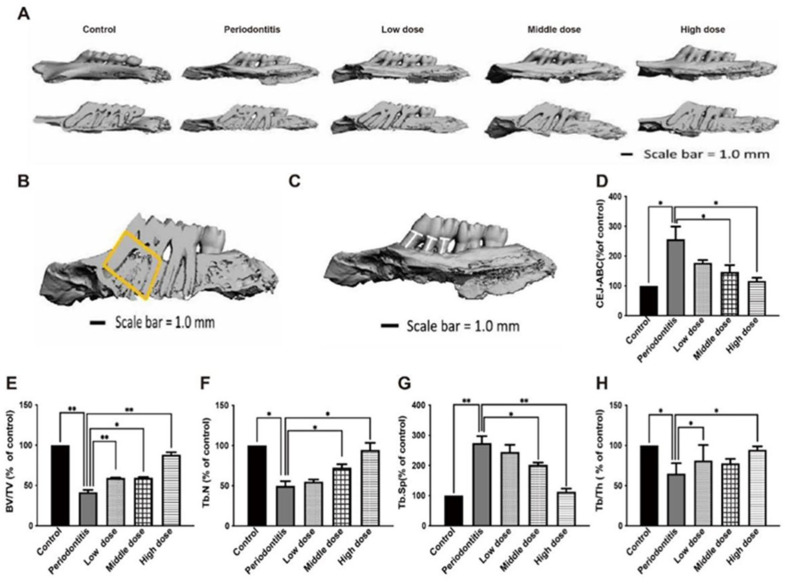
Effect of DhHP-6 on the bone microstructure around the left maxillary first molar in rats with periodontitis. (**A**) Three-dimensional reconstruction and misshapen maps of the left maxillary first molar of rats in each group. (**B**) Effect of DhHP-6 on the bone microstructure around the left maxillary first molar in rats with periodontitis. The yellow area is the region of interest (ROI) of alveolar bone selected in this study. After determining the ROI region, Micro-CT was used to analyze the bone structure parameters in this region. The parameters selected in this study included: BV/TV; Tb. Th; Tb. N; Tb. Sp. (**C**,**D**) Effect of DhHP-6 on the alveolar bone resorption of the left maxillary first molar in rats with periodontitis. The white line marks the CEJ-ABC distance. The bar chart shows the CEJ-ABC distance in each group. (**E**–**H**) Quantitative analysis of the area marked in yellow in panel B. * *p* < 0.05, ** *p* < 0.01.

Micro-CT analysis of the protective effect of DhHP-6 rats on alveolar bone resorption. To evaluate the effect of DhHP-6 on alveolar bone resorption in rats with periodontitis, we used micro-CT scanning to compare alveolar bone loss and bone structure parameters of the left maxillary first molar of each group of rats [33]. A 3D reconstruction of the left maxillary of the rats revealed that alveolar bone resorption was obvious in the periodontitis group and the low-dose DhHP-6 group. The alveolar bone resorption was significantly lower in the high-dose and middle-dose DhHP-6 groups than in the periodontitis group, indicating that high-dose DhHP-6 had a protective effect against alveolar bone resorption in rats with periodontitis (Figure 5A).

In addition, BV/TV, Tb.N, Tb.Sp, Tb.Th, and other rat alveolar bone microstructure parameters were measured. Rats in the periodontitis group had lower BV/TV, Tb.N, and Tb.Th values than those in the control group, while rats in the high dose DhHP-6 group had higher BV/TV, Tb.N, and Tb.Th values than those in the periodontitis group. Similarly, BV/TV, Tb.N, and Tb.Th values were higher in the medium-dose DhHP-6 group than in the periodontitis group, while the opposite results were obtained for Tb.Sp (Figure 5B,E–H). In summary, DhHP-6 improves the microstructure of the alveolar bone in rats with periodontitis, further supporting the therapeutic effect of DhHP-6 in periodontitis.

The linear distance from CEJ to ABC was measured. As shown in Figure 5C,D, the CEJ-ABC distance in the periodontitis group was significantly higher than that in the blank control group, while the CEJ-ABC distance in the high-dose DhHP-6 group was significantly lower than that in the periodontitis group. In addition, the CEJ-ABC distances in the low-dose DhHP-6 group and middle-dose DhHP-6 group were significantly lower than that in the periodontitis group. These results showed that DhHP-6 has a protective effect on alveolar bone resorption in rats with periodontitis.

### 3.6. In Vivo Biosafety of DhHP-6

**Figure 6:**
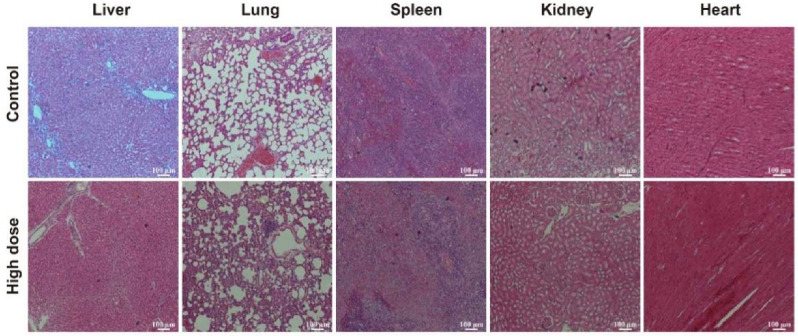
H&E staining showing the effects of high-dose DhHP-6 on the heart, lung, liver, spleen, and kidney of rats. Scale bars represent 100 μm.

DhHP-6 does not damage rat organs. H&E staining was used to observe the morphological and histological changes in rat organs (Figure 6) [34]. Compared with observations in the control, there were no obvious morphological and histological changes in the liver, lung, spleen, kidney, heart, and other main organs in the high-dose DhHP-6 group. These results indicated that DhHP-6 has good biocompatibility and biosafety and does not exert toxic effects on the main organs of rats.

## 4. Discussion

Periodontitis is a chronic inflammatory disease. If not treated early, it will damage the hard tissues around the teeth [35]. Many natural peroxidases and peroxidase-mimicking enzymes have bactericidal and anti-inflammatory effects [22]. In addition, many polypeptide drugs, such as bee venom, metformin, resveratrol, and proanthocyanidins, have good anti-inflammatory effects [36,37,38,39]. DhHP-6 is a new synthetic peroxidase-mimicking enzyme with good peroxidase activity, antioxidant effects, and the ability to scavenge free radicals. It also has the potential to act as an antioxidant enzyme [23,24].

A large amount of ROS is produced in gingival lesions of patients with periodontitis, resulting in a redox imbalance and oxidative stress, eventually leading to the destruction of gingival tissue [40]. Recent studies have shown that an imbalance between the antioxidant enzyme defense system, ROS, proteolytic enzymes, and oxidants is the main cause of periodontal tissue damage [41]. Under normal conditions, intracellular antioxidant enzymes and antioxidants can neutralize ROS produced during cell metabolism; however, excessive ROS weakens the antioxidant capacity of SOD and CAT, thus increasing the consumption of intracellular SOD, CAT, and the non-enzymatic antioxidant GSH, making cells vulnerable to attack by oxidants and resulting in tissue damage [42,43,44]. GSH, SOD, and CAT, as the main antioxidant enzymes [45], can clear accumulated ROS and reduce cell and tissue damage caused by oxidative stress [46,47]. The use of antioxidants to clear intracellular ROS and reduce intracellular oxidative stress has become an important method for the treatment of periodontitis [48]. Due to its peroxidase activity and antioxidant capacity, DhHP-6, a peroxidase-mimicking enzyme, reduced ROS production in HGFs under oxidative stress. Our results show that DhHP-6 has the potential to act as an antioxidant enzyme, increase levels of CAT and GSH in HGFs, reduce the production of the intracellular oxidation product MDA, eliminate excess intracellular ROS, reduce intracellular ROS accumulation, increase the antioxidant capacity of HGFs, and reduce oxidative damage in HGFs. At the same time, DhHP-6 significantly reduced the rate of apoptosis of HGFs induced by H_2_O_2_, effectively attenuated the symptoms of periodontitis, and slowed disease development.

TNF-α, IL-1β, and IL-6 levels in the gingival crevicular fluid of patients with periodontitis are significantly higher than those in healthy individuals, and these cytokines decreased significantly after treatment. Therefore, the proinflammatory cytokines TNF-α, IL-1β, and IL-6 play a key role in the occurrence and development of periodontitis. Previous studies have shown that DhHP-6 has anti-inflammatory effects as a peroxidase-mimicking enzyme. Our results indicated that DhHP-6 has therapeutic effects in periodontitis by reducing the levels of the inflammatory factors TNF-α and IL-1β in HGFs sensitized by LPS and reducing the cellular inflammatory response [29,49].

The tissue structure of the oral gingival epithelium and conjunctival epithelium of rats is similar to that of humans; therefore, rats are a good choice for experimental studies. There are many methods to establish a rat model of periodontitis, including the intragastric administration of bacteria, LPS smearing, and silk thread ligation [50]. We established a rat model of acute periodontitis induced by ligation and verified successful model establishment based on clinical diagnostic criteria. After successful modeling, the changes in gingival inflammation, antioxidant factors, pro-inflammatory cytokines, ROS production, and alveolar bone resorption in rats supported the therapeutic effect of DhHP-6 on periodontitis. Our results show that DhHP-6, as a peroxidase-mimicking enzyme, has antioxidant properties and can scavenge free radicals. By increasing the expression levels of GSH, SOD, and CAT, DhHP-6 exerts antioxidation effects.

## 5. Conclusions

In summary, we have demonstrated that the DhHP-6 peroxidase-mimicking enzyme material exhibits synergistic antioxidant and anti-inflammatory effects in either an LPS-induced inflammatory model or an animal model of periodontitis in rats. The regular injection of DhHP-6 reduces oxidative damage in periodontal tissues. Therefore, DhHP-6 is a promising new peroxidase biomimetic material for the treatment of periodontitis and the prevention of periodontal disease. However, there are currently no clinically effective antioxidants for the treatment of periodontitis, and in this study DhHP-6 was not compared to another antioxidant compound; therefore, we are unable to determine its relative effectiveness and consequently its therapeutic potential. A number of studies have confirmed the effect of antioxidants on periodontitis as a complementary treatment modality, but there is still a lack of data that support the long-term effects of DhHP-6 in periodontitis as a chronic progressive disease, which will be the focus of our next studies.

## Data Availability

The datasets used and analyzed during the current study are available from the corresponding author upon reasonable request. All data generated during this study are included in this published article.
